# Lycopene inhibits pyroptosis of endothelial progenitor cells induced by ox-LDL through the AMPK/mTOR/NLRP3 pathway

**DOI:** 10.1515/med-2024-0973

**Published:** 2024-06-24

**Authors:** Chujun Tan, Junqiu Chen, Tengcan Tu, Lifang Chen, Jun Zou

**Affiliations:** The Second School of Clinical Medicine, Southern Medical University, Guangzhou, 510280, China; Department of Cardiology, Fuwai Hospital, Chinese Academy of Medical Sciences, (Shenzhen Sun Yat-sen Cardiovascular Hospital), Shenzhen, 518057, China; Department of Cardiology, Shenzhen Qianhai Shekou Free Trade Zone Hospital, Shenzhen, 528200, China; Department of Cardiology, The Sixth Affiliated Hospital, School of Medicine, South China University of Technology, Foshan, 528200, China

**Keywords:** lycopene, AMPK/mTOR, NLRP3, ox-LDL, EPCs

## Abstract

The malfunction of endothelial progenitor cells (EPCs) due to ox-LDL is a risk contributor for arteriosclerotic disease. Meanwhile, lycopene possesses anti-inflammatory and antioxidative qualities. This investigation aimed to determine if lycopene can protect EPCs from ox-LDL-induced damage and to elucidate the underlying mechanism. The effects of lycopene on the survival, migration, and tube-forming capacity of EPCs were determined via *in vitro* assays. Expression of proteins related to pyroptosis and cellular proteins related to AMPK/mTOR/NLRP3 signaling was determined by western blot/flow cytometry. Our results demonstrated that lycopene treatment significantly enhanced proliferation, tube formation, and migration of EPCs stimulated by ox-LDL. Additionally, lycopene was found to suppress pyroptosis in ox-LDL-induced EPCs through the activation of AMPK, which led to the inhibition of mTOR phosphorylation and subsequent downregulation of the downstream NLRP3 inflammasome. In summary, our study suggests that lycopene mitigates ox-LDL-induced dysfunction in EPCs and inhibits pyroptosis via AMPK/mTOR/NLRP3 signaling. Our study suggests that lycopene may act as promising therapies for preventing atherosclerosis.

## 1 Introduction

Atherosclerotic cardiovascular disease (ASCVD) continues to cause illness and death worldwide, as indicated by multiple studies [1], and it is well-established that low-density lipoprotein (LDL) is a contributing factor for ASCVD development [2]. During oxidative stress, LDL undergoes modification, leading to the formation of oxidized LDL (ox-LDL), a crucial atherogenic alteration of native LDL. The initial occurrence of LDL oxidation within the vascular endothelium has been documented as a crucial step in the genesis of atherosclerotic plaques [3,4]. Endothelial dysfunction stands as a primary mechanism implicated in the development of arteriosclerotic disease. Recent evidence has suggested an association between endothelial dysfunction and dysfunction of endothelial progenitor cells (EPCs) [5,6]. EPCs, which are released from the bone marrow into the peripheral blood, demonstrate significant involvement in vascular regeneration, endothelial repair, and the substitution of dysfunctional endothelium [7]. This is achieved through their incorporation into the injured vessel site, differentiation into endothelial cells, and secretion of paracrine [7]. EPCs can migrate to distant blood vessels and undergo differentiation into fully developed endothelial cells, effectively replacing aged or damaged endothelial cells. The effectiveness of this repair process relies on the quantity and functionality of EPCs [6]. Furthermore, it has been observed that ox-LDL significantly influences EPC activities through the LOX-1/NLRP3 inflammasome pathway, leading to cellular senescence and apoptosis [5]. However, a precise understanding of the molecular mechanisms through which ox-LDL affects EPCs remains elusive.

Inflammatory responses and their mediators are recognized to modulate atherosclerosis (AS). In particular, NLRP3, a cytosolic pattern recognition receptor, has garnered significant attention from researchers due to its ability to bind to detrimental factors [8]. For instance, NLRP3 inflammasomes have been reported to accumulate at cleavage sites, thereby stimulating interleukin (IL)-1β and caspase-1 activities, leading to pyroptosis [9] and hastening the inflammatory process in AS [5]. Additionally, the AMP-activated protein kinase (AMPK) acts as an intracellular energy controller and detector for various metabolic functions. On the other hand, mTOR is a downstream target of AMPK and has been implicated in protein synthesis, lipid biosynthesis, cell growth maintenance, and atherogenesis [10,11]. Intriguing findings have shown that ox-LDL induces autophagy through AMPK/mTOR signaling in macrophages [12] as well as HUVECs [13]. Meanwhile, NLRP3 inflammasomes were found to be activated by inhibiting AMPK/mTOR signaling in cardiomyocytes exposed to high glucose [14]. However, the connection between AMPK/mTOR and NLRP3 signaling in ox-LDL-stimulated EPCs remains unclear.

Intriguingly, lycopene is a red carotenoid known for its antioxidative and anti-inflammatory properties, which help prevent cardiovascular diseases, including hyperlipidemia and atherosclerosis [15,16,17,18]. A recent study has shown that lycopene promotes the survival of EPCs and protects them from apoptosis and oxidative autophagy induced by advanced glycation end products [19]. Additionally, lycopene has been indicated to promote autophagy [20] and inhibit inflammation [21] by activating AMPK. In addition, the anti-oxidative stress has been demonstrated in various studies [22,23,24,25]. Lycopene could alleviate oxidative stress-induced cell injury in human vascular endothelial cells by encouraging the SIRT1/Nrf2/HO-1 pathway [26]. Additionally, lycopene enhanced acyl-platelet-activating factor biosynthesis in endothelial cells during oxidative stress [27]. Lycopene could rescue S phase of the cell cycle arrest, reduced apoptosis rate, and decreased autophagic reaction including reactive oxygen species and mitochondrial membrane potential of EPCs under diabetic conditions [19]. However, whether lycopene offers protection against ox-LDL-induced EPC dysfunction and specific underlying mechanisms still require clarification. Consequently, this study was undertaken to evaluate lycopene’s protective impact on ox-LDL-induced EPC damage and to unveil mechanisms through which lycopene influences the AMPK/mTOR/NLRP3 signaling in an *in vitro* setting.

## 2 Materials and methods

### 2.1 EPC isolation

EPCs were isolated and cultured following established procedures [7] and were sourced from San Yat-sen Memorial Hospital in Guangzhou, China. To summarize the process, peripheral blood mononuclear cells were separated via density gradient centrifugation. Following this, the cells underwent three washes with D-Hank’s buffer before being introduced into endothelial cell medium (ScienCell Research Laboratories, USA) supplemented with 10% fresh fetal bovine serum. The cells were passaged when they reached 70–80% confluence. Late-stage cells that had undergone three passages and were cultured for 28 days were utilized in subsequent experiments.

### 2.2 EPCs characterization

The late-stage cultured cells were treated with 1,1′-dioctadecyl-3,3,3′,3′-tetramethylindocarbocyanine-acetylated low-density lipoprotein (DiI-Ac-LDL) at a concentration of 2.4 μg/mL (Solarbio, China) for a duration of 1 h at 37°C. Afterwards, they were rinsed with phosphate-buffered saline (PBS), fixed using 4% polyoxymethylene (POM) (Beyotime, China) for 10 min, and subsequently incubated with fluorescein isothiocyanate (FITC)-labeled UEA-1 at a concentration of 10 μg/mL (Sigma-Aldrich, USA) for 1 h at 37°C.

To complete the process, the cells were given another rinse and were then subjected to incubation with a glycerol reagent. Imaging was carried out using fluorescence microscopy (Zeiss Smartproof 5, Germany). In this context, red and green fluorescent cells signified Dil-Ac-LDL uptake and the binding of UEA-1, respectively. Cells that displayed both red and green fluorescence confirmed the presence of late EPCs.

### 2.3 Immunofluorescence staining

To further evaluate the expression of late EPCs, cultured EPCs were fixed using a 2% POM. Subsequently, the cells underwent another round of PBS washing and were blocked with 10% PBS at room temperature for 30 min. Next, primary antibodies including CD31 (11265-1-AP, 1:100, Wuhan, China), CD34 (ab81289, 1:100, Abcam, Cambridge, UK), CD133 (ab19898, 1:100, Abcam), and CD45 (ab33923, 1:100, Abcam) were applied to the cells and incubated overnight at 4°C. Following this, cells were incubated with secondary antibodies for 1 h at 37°C, and the cell nuclei were counterstained with 4′,6-diamidino-2-phenylindole (Beyotime, Beijing, China). Finally, immunofluorescence staining was assessed using a fluorescence microscope.

### 2.4 Treatments

For the concentration-dependent studies, EPCs were exposed to ox-LDL (0, 25, 50, 100 μg/mL) or lycopene (0, 10, 25, 50 μg/mL) (S24983; Shanghai Yuanye Bio-Technology Co., Ltd., Shanghai, China) for 24 or 48 h before subsequent assays. In some studies, EPCs were treated with 100 µg/mL ox-LDL for 24 h followed by treatment with lycopene (100 μg/mL) for 24 h before subsequent assays. In the studies involving AMPK inhibitor, compound C (CC), EPCs were treated with 100 µg/mL ox-LDL for 24 h followed by pretreatment CC (1 μg/mL) for 30 min and were then treated with lycopene (100 μg/mL) for another 24 h.

### 2.5 Cell viability assay

The cell counting kit-8 (CCK-8) assay (Beyotime, China) was utilized to determine the optimal concentrations of ox-LDL or lycopene and assess cell viability. In brief, EPCs were exposed to different treatments. Following the treatments, CCK-8 solution was added to each well and cells were incubated with CCK-8 solution for 2 h at 37°C. Cell viability was determined by measuring absorbance at 450 nm using a microplate reader.

### 2.6 Wound healing assay

A total of 1.6 × 10^4^ EPCs were seeded into each well of a 12-multiwell dish (NEST Biotech, China). Once the cells adhered to the bottom of the well, the cell layer was scratched using a 1,000 μL pipette tip and incubated at 37°C with 5% CO_2_. Wound closure was observed under a microscope at 0, 24, and 48 h, respectively. Cell images were captured, and the wound closure was quantified using ImageJ software.

### 2.7 Tube formation

After treating the cells with various conditions for 48 h, EPCs were suspended in serum-free culture medium to achieve a concentration of 4 × 10^5^ cells/mL, and 100 μL cell suspension was added to each well. Plates were then incubated at 37°C with 5% CO_2_ for 24 h and examined under a microscope at 6, 24, and 48 h. Quantitative analysis of tubule length was conducted using ImageJ software.

### 2.8 Flow cytometry

Pyroptosis was assessed via flow cytometric analysis using propidium iodide (#ab14083; Abcam, UK), and an active caspase-1 antibody (#89332; Cell Signaling Technology, USA). To identify the cells expressing these markers, EPCs were incubated for 30 min at 4°C with PE-labeled antibody or corresponding IgG isotype control. More than 5 × 10^6^ EPCs were collected for flow cytometry analysis (MoFlo Astrios, Beckman-Coulter, USA).

### 2.9 Western blot

EPCs were lysed in cell lysis buffer (#A3678-25G; Sigma-Aldrich, USA) supplemented with protease and phosphatase inhibitor cocktails (Thermo Fisher Scientific). Proteins were separated using sodium dodecyl sulfate-polyacrylamide gel electrophoresis and then transferred to a polyvinylidene fluoride membrane (Servicebio, China). After blocking with 5% milk, the membranes were incubated with the following antibodies: NLRP3 (#ab263899; 1:1,000, Abcam), GSDMD-N (#36425; 1:1,000, CST), caspase-1 (#89332; 1:1,000, CST), IL-1β (#83186; 1:1,000, CST), IL-18 (#ab207324; 1:200, Abcam), AMPK (#ab131512; 1:1,000, Abcam), mTOR (#ab32028; 1:2,000, Abcam), p-AMPK (#ab23875; 1:1,000, Abcam), p-mTOR (#ab109268; 1:500, Abcam), and β-actin (#GB11001; 1:2,000, Servicebio, China). Finally, secondary antibodies (#HA1001; 1:5,000, HuaBio, China) were applied, and the signal intensities were quantified using ImageJ software.

### 2.10 Statistical analysis

Statistical analyses were conducted using SPSS software (version 25.0, IBM SPSS) and R version 4.0.2. Data is presented as the mean ± standard deviation. Data normality was performed using Kolmogorov–Smirnov test. Group comparisons were carried out using one-way ANOVA followed by Bonferroni’s post hoc test with 95% confidence intervals. *P*-value <0.05 was regarded as statistically significant. Each group included three biological replicates.


**Ethical approval:** The experiments were carried out in accordance with the Declaration of Helsinki of the World Medical Association and the protocol was approved by the Ethics Committee of The Sixth Affiliated Hospital, School of Medicine, South China University of Technology (No. 2021-108).

## 3 Results

### 3.1 Characterization of EPCs

**Figure A1:**
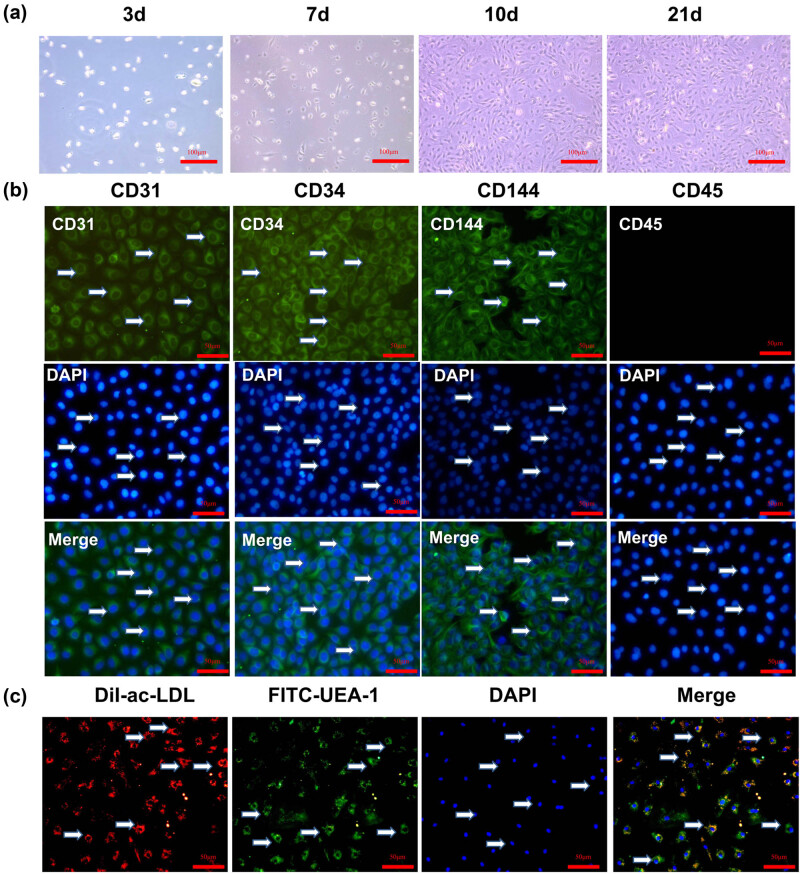
Characterizations of EPCs. (a) The basic morphological changes of EPCs at different time points (3, 7, 10 and 21 d) under a microscope; (b) Immunofluorescence was used to detect the expression of EPCs markers (CD31+/CD34+/CD45−/CD133+). (c) The Dil-ac-LDL and FITC-UEA-1 double staining was used to identify the endothelial function of EPCs. All the experiments have three biological repeats (*n* = 3).

EPCs were classified into two distinct types: early and late EPCs. Early EPCs appear after 5–7 days, and late EPCs appear after 14–21 days [28]. Late EPCs exhibited typical spindle morphology seven days after culturing (Figure A1a). Our results from laser confocal microscopy, utilizing a co-staining technique with Dil-Ac-LDL/FITC-UEA-1, confirmed that these cells belonged to endothelial lineage, i.e., EPCs (Figure A1c). Further characterization of EPCs was based on their ability to differentiate and their expression of CD34+/CD31+/CD133+/CD45−, as confirmed by immunofluorescence staining (Figure A1b) [29]. Therefore, these EPCs were selected for subsequent experiments.

### 3.2 Lycopene prevents ox-LDL-induced dysfunction in EPCs

**Figure A2:**
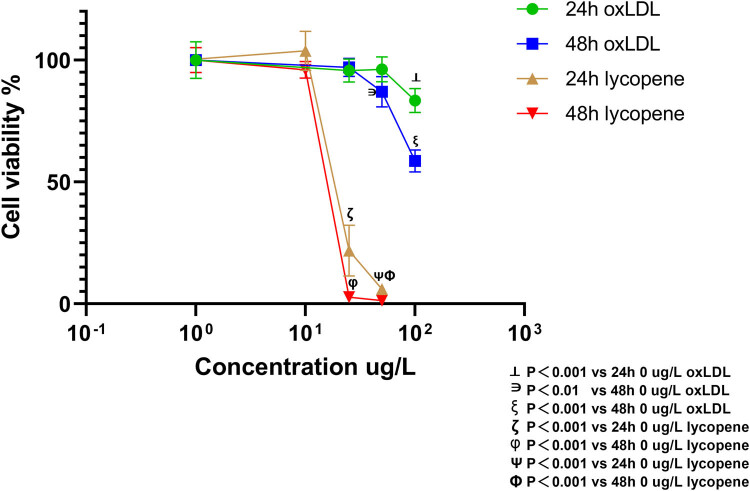
Cell viability of EPCs treated with different concentrations of ox-LDL and Lyc. CCK-8 was used to detect the cell viability of EPCs cells treated with different concentrations of ox-LDL (0, 25, 50, 100 μg/mL) and Lyc (0, 10, 25, 50 μg/mL) for 24 and 48 h. All the concentrations in this figure adopted log form. All the experiments have three biological repeats (*n* = 3).

We initially examined the impact of various concentrations of ox-LDL on EPC proliferation using a CCK-8 assay. From these results, we identified the most suitable concentration of ox-LDL (100 µg/mL) and treatment duration (48 h) for subsequent experiments (Figure A2). Importantly, we observed that EPC viability was not adversely affected by treatment with 10 µg/mL lycopene for either 24 or 48 h compared to the control group (see Figure A2). Consequently, we opted to use 10 µg/mL lycopene for a 24-h duration in the subsequent experiments.

**Figure 1:**
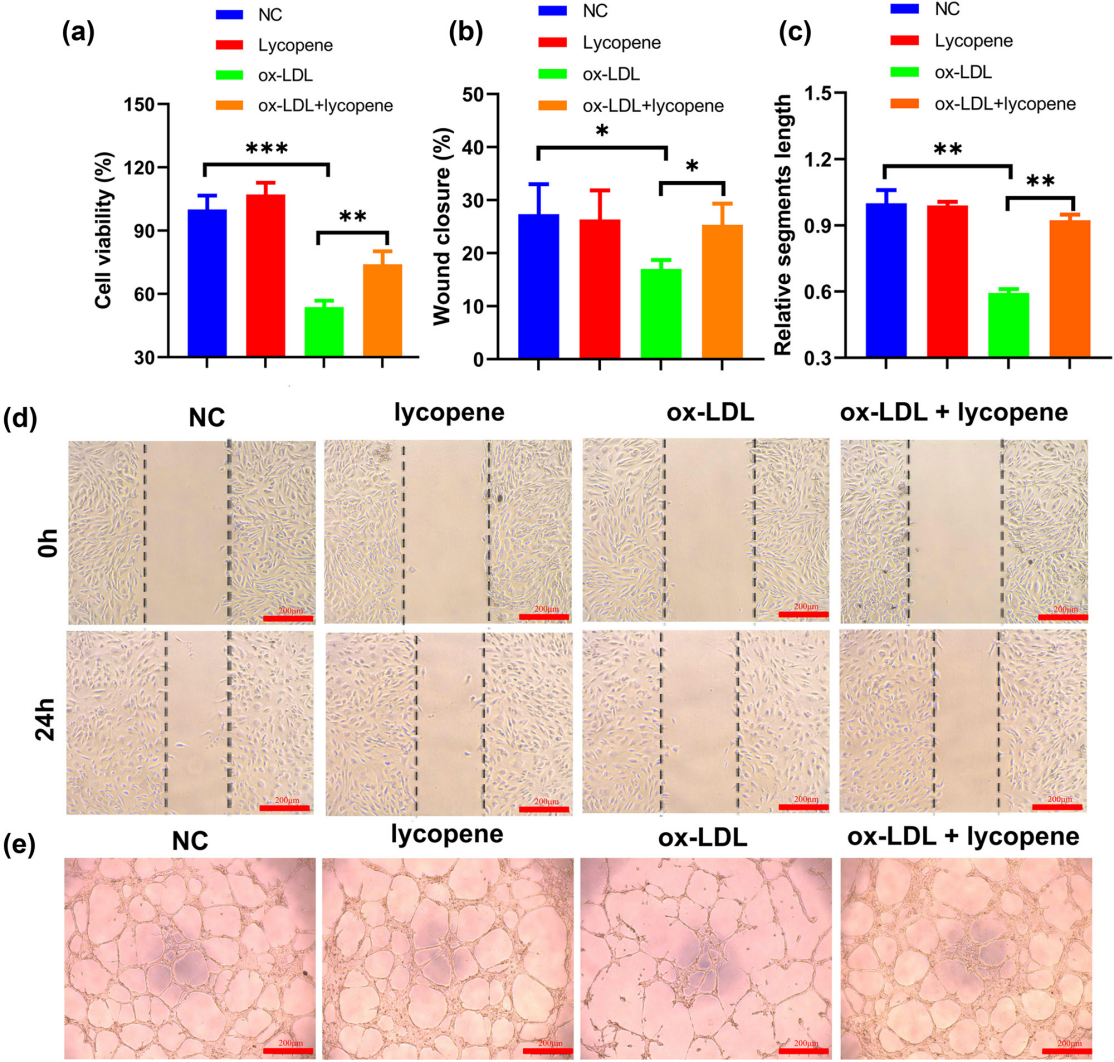
Lycopene prevents ox-LDL-induced dysfunction in EPCs. (a) CCK-8 was used to detect the effect of Lyc on the activity of ox-LDL-induced EPC cells. (b and d). Scratch test was used to evaluate the effect of Lycopene on ox-LDL-induced EPC migration. (c and e) Tube formation assay was used to evaluate the effect of Lycopene on ox-LDL-induced EPC angiogenesis. All the experiments have three biological repeats (*n* = 3). *P < 0.05, **P < 0.01, ***P < 0.001.

Furthermore, our findings showed that 10 µg/mL lycopene exhibited protective actions in ox-LDL-induced cell viability (Figure 1a). In the wound healing assay, we observed a significantly accelerated rate of wound healing in ox-LDL-treated EPCs with lycopene treatment compared to ox-LDL-treated cells (see Figure 1b and d). Additionally, the tube formation assay revealed a heightened angiogenic capacity in ox-LDL-treated EPCs with lycopene treatment in comparison to ox-LDL-treated cells (see Figure 1c and e). In summary, these results collectively indicate that lycopene contributes to the restoration of EPC functionality impaired by ox-LDL.

### 3.3 Suppression of pyroptosis in EPCs by lycopene

**Figure 2:**
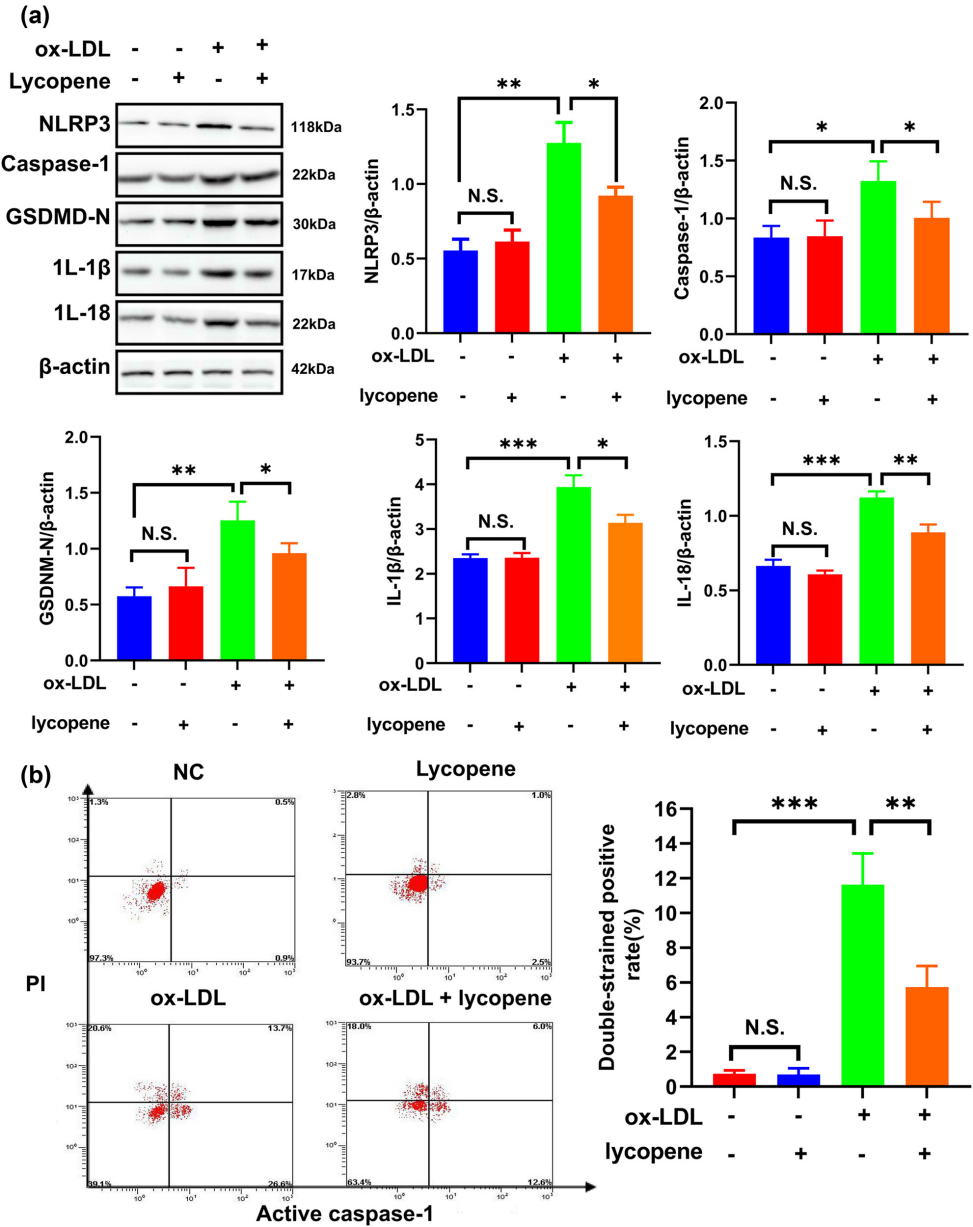
Lycopene inhibits the pyroptosis of EPCs induced by ox-LDL. (a) Western blot was used to detect the expression of pyroptosis-related proteins (NLRP3, caspase-1, GSDMD-N, IL-1β, IL-18). (b) Flow cytometry was used to detect the cell pyroptosis. All the experiments have three biological repeats (*n* = 3). *P < 0.05, **P < 0.01, ***P < 0.001.

We further employed a western blot assay to examine pyroptosis-related protein expression. Our findings revealed a substantial increase in these protein levels in the ox-LDL-treated EPCs when compared to the control group (Figure 2a). However, following the introduction of lycopene, the expression of these proteins notably decreased in ox-LDL-treated EPCs with lycopene treatment as opposed to ox-LDL-treated cells (Figure 2a). Results from flow cytometry indicated that pyroptosis rate in ox-LDL-treated EPCs with lycopene treatment was significantly lower than that in ox-LDL-treated cells (Figure 2b). These observations suggest that lycopene holds the potential to suppress ox-LDL-induced pyroptosis.

### 3.4 Lycopene inhibits ox-LDL-induced cell damage via AMPK/mTOR

**Figure 3:**
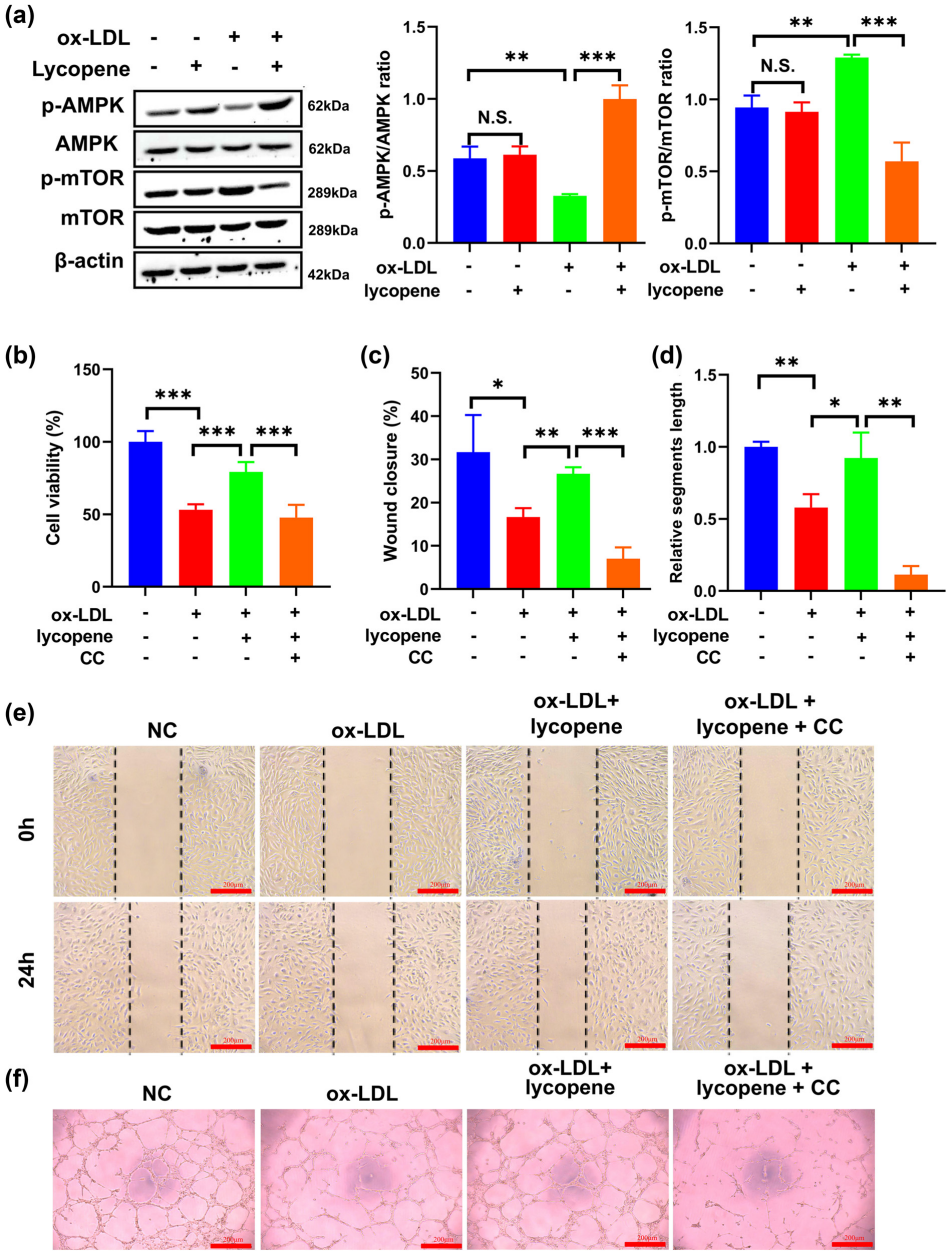
Lycopene regulates ox-LDL-induced EPC proliferation, migration, and tube formation by activating the AMPK/mTOR signaling pathway. (a) Western blot was used to detect the effect of Lycopene on the AMPK/mTOR signaling pathway in ox-LDL-induced EPCs cells. (b) CCK-8 was used to detect the effect of Lycopene on the cell viability of ox-LDL-induced EPCs after adding AMPK inhibitor, compound C (CC). (c and e) Scratch test was used to detect the effect of Lycopene on the migration ability of ox-LDL-induced EPCs after adding an AMPK inhibitor, compound C (CC). (d and f) Tube formation experiment was used to detect the effect of Lycopene on the tube formation ability of ox-LDL-induced EPCs after adding an AMPK inhibitor, compound C (CC). All the experiments have three biological repeats (*n* = 3). *P < 0.05, **P < 0.01, ***P < 0.001.

To uncover the mechanisms by which lycopene mitigates ox-LDL-induced damage in EPCs, we conducted an investigation into AMPK/mTOR signaling, a pivotal component in pyroptosis. Among the four groups, AMPK and mTOR protein levels did not exhibit significant differences (Figure 3a).

However, it is noteworthy that in the ox-LDL-treated EPCs, there was an increase in p-mTOR level in comparison to the control group (Figure 3a). Conversely, p-AMPK expression showed a decrease in the ox-LDL-treated EPCs compared with control (Figure 3a). Interestingly, in ox-LDL-treated EPCs with lycopene treatment, the p-mTOR level was notably reduced, and the p-AMPK level was elevated compared with ox-LDL-treated cells (Figure 3a). These initial findings indicated that lycopene was capable of hindering cell damage induced by ox-LDL through the AMPK/mTOR pathway.

### 3.5 Involvement of AMPK/mTOR signaling in late EPCs’ *in vitro* capacity

To further confirm the involvement of AMPK/mTOR, we introduced a specific AMPK inhibitor (CC) and evaluated several endpoints. Cell viability was noticeably reduced in ox-LDL-treated EPCs with lycopene and CC treatment comparing ox-LDL-treated cells with lycopene treatment (Figure 3b). Wound healing assays demonstrated a decrease in the average rate of wound healing in ox-LDL-treated EPCs with lycopene and CC treatment in contrast to ox-LDL-treated cells with lycopene treatment (Figure 3c and e). Likewise, the tube formation assay revealed diminished angiogenic capabilities in ox-LDL-treated EPCs with lycopene and CC treatment compared with ox-LDL-treated cells with lycopene treatment (Figure 3d and f).

**Figure 4:**
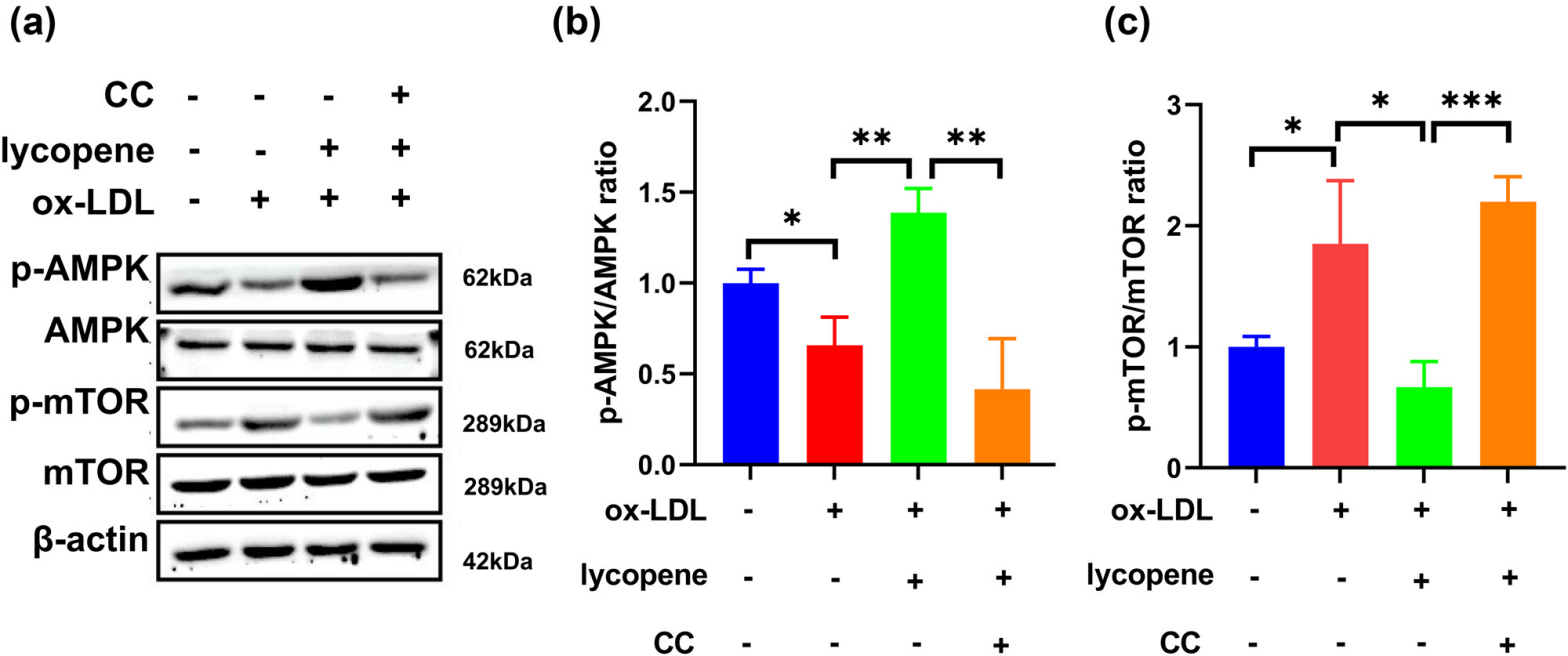
CC inhibits the activation of Lycopene on the AMPK/mTOR signaling pathway of EPCs induced by ox-LDL. (a) Western blot was used to detect the effect of Lycopene on the AMPK/mTOR signaling pathway in ox-LDL-induced EPCs s after adding an AMPK inhibitor, compound C (CC). (b) p-AMPK expression level. (c) mTOR expression level. All the experiments have three biological repeats (*n* = 3). *P < 0.05, **P < 0.01, ***P < 0.001.

Western blot analysis highlighted that in ox-LDL-treated EPCs with lycopene and CC treatment, p-mTOR level was significantly elevated, while p-AMPK level was significantly decreased when contrasted with ox-LDL-treated cells with lycopene treatment (Figure 4a–c). These findings suggest that CC, acting as an inhibitor of AMPK, counteracts the effects of lycopene on EPC dysfunction induced by ox-LDL. Activation of the AMPK/mTOR signaling pathway could be attributed to the phosphorylation of AMPK.

### 3.6 Lycopene inhibits pyroptosis in EPCs via AMPK/mTOR signaling

**Figure 5:**
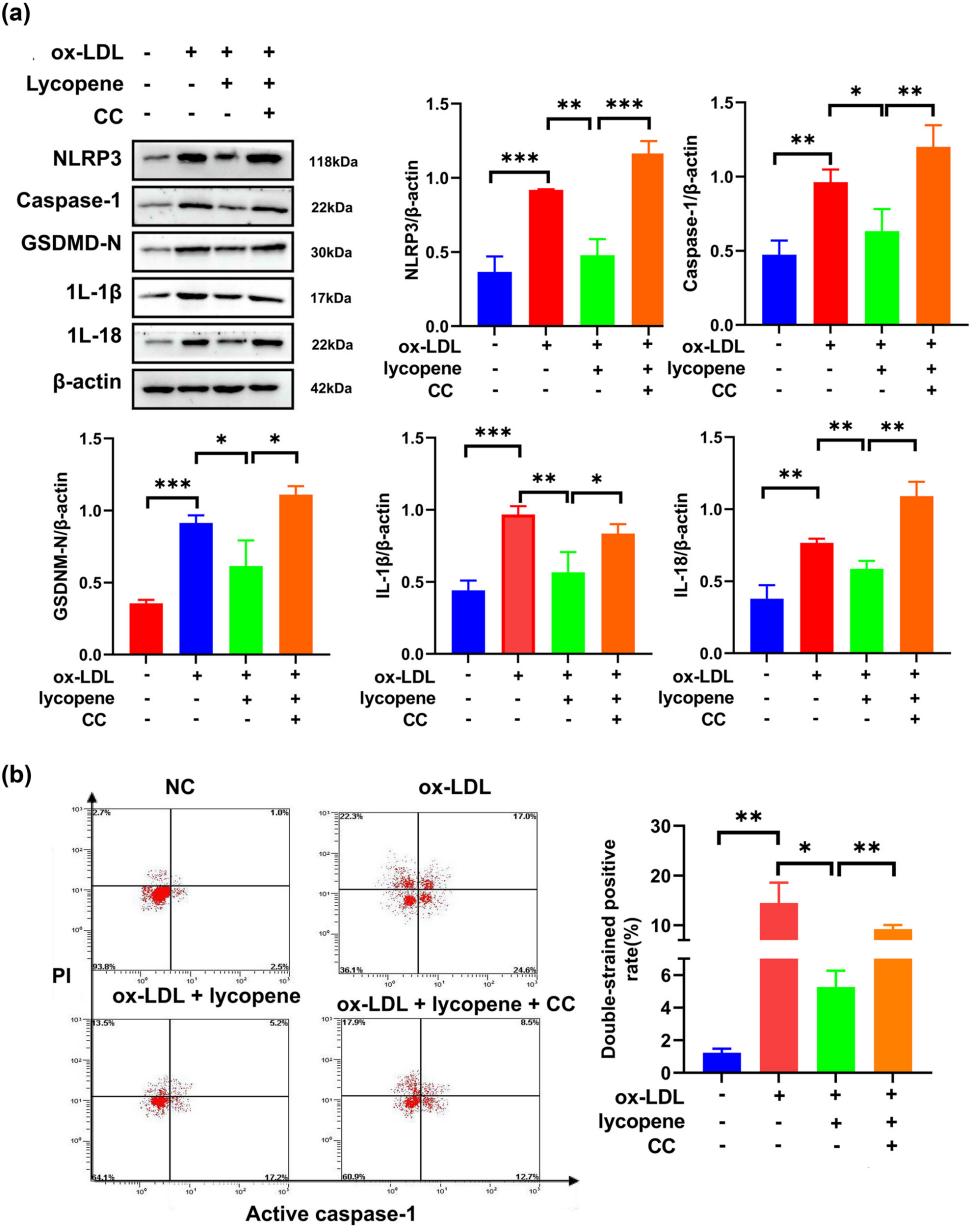
Lycopene inhibits EPCs pyroptosis induced by ox-LDL by activating the AMPK/mTOR/NLRP3 signaling pathway. (a) Western blot was used to detect the expression of pyroptosis-related proteins (NLRP3, caspase-1, GSDMD-N, IL-1β, IL-18). (b) Flow cytometry was used to detect the cell pyroptosis. All the experiments have three biological repeats (*n* = 3). *P < 0.05, **P < 0.01, ***P < 0.001.

After confirming lycopene’s activation of AMPK, we proceeded to investigate the impact of CC on pyroptosis-related proteins. Expression levels of caspase-1, NLRP3, IL-1β, GSDMD-N, and IL-18 were significantly diminished in LDL-treated cells with lycopene treatment in comparison to ox-LDL-treated cells (Figure 5a). However, the introduction of CC antagonized the anti-pyroptosis effect of lycopene, resulting in a notable increase in the levels of pyroptosis-associated proteins (Figure 5a).

Flow cytometry yielded consistent findings: in contrast with ox-LDL-treated EPCs, pyroptosis rate in the ox-LDL-treated cells with lycopene treatment was notably reduced. Nonetheless, the incorporation of CC into the ox-LDL-treated EPCs with lycopene treatment resulted in a substantial increase in pyroptosis rate (Figure 5b). In conclusion, these results indicated that lycopene activates AMPK to inhibit EPC pyroptosis induced by ox-LDL through phosphorylated AMPK repression of mTOR/NLRP3.

## 4 Discussion

Our findings indicate that lycopene inhibited pyroptosis and enhanced the viability, tube formation, and migration of EPCs exposed to ox-LDL through the AMPK/mTOR/NLRP3 axis. This effect was validated by the addition of CC (a selective AMPK inhibitor). It is widely recognized that AS is a chronic condition affecting vessel walls characterized by the development of lipid-rich plaques. Ox-LDL exacerbates plaque formation by escalating oxidative stress and endothelial damage while impeding the restoration of impaired endothelium [30]. Endothelial apoptosis triggered by ox-LDL, LPS, and methylglyoxal has also been reported to contribute to the progression of endothelial dysfunction in AS [31,32,33]. EPCs possess the ability to differentiate into mature endothelial cells and play a role in both physiological and pathological neovascularization. This function is crucial for maintaining a dynamic equilibrium between endothelial repair and injury [34]. Ox-LDL-induced EPC dysfunction has been reported by Ji et al. [35]. Moreover, Yang et al. reported that ox-LDL inhibited EPC proliferation and activated protective autophagy response by promoting calcium entry and reducing downstream mTOR phosphorylation [36]. Excessive intake of ox-LDL could upregulate caspase-1, NLRP3, and IL-1 in epithelial cells, suggesting an increase in pyroptosis. Consistent with prior research, our findings confirm that ox-LDL impairs the proliferation, migration, and tube formation ability of EPCs and induces EPC pyroptosis.

Recently, a systematic review and meta-analysis using flow-mediated dilation demonstrated that lycopene supplementation could improve endothelial function [37]. Another meta-analysis showed that lycopene can reduce the risk of cardiovascular diseases by 17% when comparing the highest reported exposure to the lowest exposure [38]. However, whether lycopene could attenuate the decline in cell function induced by ox-LDL remains unclear. Our study suggests that lycopene significantly enhances viability, tube formation, and migration of ox-LDL-treated EPCs. Moreover, a study using a mouse model demonstrated that lycopene alleviates di(2-ethylhexyl)phthalate-induced pyroptosis in the spleen by suppressing caspase-1/NLRP3 [39]. Additionally, lycopene has been shown to suppress NF-κB activation and the expression of adhesion molecules through Nrf2-mediated heme oxygenase-1 in endothelial cells [40]. Lycopene has also been indicated as an AMPK activator with an anti-neuroinflammatory effect [21]. In our study, we observed that lycopene treatment activated AMPK and inhibited mTOR phosphorylation, leading to the downregulation of the NLPR3 inflammasome and caspase-1. This ultimately resulted in the inhibition of cell pyroptosis in EPCs, which are vulnerable to injury by ox-LDL.

Pyroptosis has been reported to be executed by inflammasomes, with the most common inflammasome being NLRP3 [8]. Furthermore, the NLRP3-mediated pathway has been indicated as the main mechanism of endothelial cell pyroptosis in the initiation of AS [41]. AMPK is a key mediator in cellular energy metabolism. A study by Zha et al. found that AMPK signaling regulated ATP-induced inflammasome activation and pyroptosis [42]. Additionally, Arab et al. found that saxagliptin protected the gastric mucosa from ethanol-evoked gastropathy through NLRP3 inflammasome inhibition and AMPK/mTOR-driven autophagy activation [43]. Furthermore, AMPK has the capability to activate TSC1/2 while inhibiting mTORC1, thereby fostering autophagy [44]. It is plausible that lycopene might modulate AMPK/mTOR signaling during oxidative stress owing to its antioxidative properties [24]. Moreover, the NLRP3 inflammasome, a complex comprising the NLRP3 receptor, ASC adapter, and pro-caspase-1, plays a pivotal role in atherosclerosis development by catalyzing the maturation of caspase-1, which in turn generates IL-1β/IL-18 from pro-IL-1β/IL-18 [45]. Additionally, lycopene has been shown to mitigate chronic stress-induced hippocampal microglial pyroptosis by suppressing the NLRP3 signaling pathway [46]. Lycopene also exhibits potential in alleviating hepatic ischemia–reperfusion injury by inhibiting the NLRP3 inflammasome through the Nrf2/HO-1 pathway in Kupffer cells [47]. Consistently, our study also confirmed that lycopene activated AMPK to suppress mTOR phosphorylation and activate caspase-1 and NLRP3 inflammasome. CC treatment in this study counteracted the protective effects on EPCs pyroptosis and *in vitro* capacities. These data suggest that the AMPK/mTOR and NLRP3 inflammasome pathways form a signaling axis through which lycopene protects against ox-LDL-induced EPC dysfunction and pyroptosis.

In the present study, several limitations should be considered. First, our study elucidates a novel mechanism underlying the protective actions of lycopene on EPCs. However, the protective effect of lycopene on EPC pyroptosis may only manifest in a pyroptotic state under pathological stimuli, as our study elucidated that lycopene alone had a limited action on the AMPK/mTOR/NLRP3 axis. Second, the present study only examined the AMPK/mTOR/NLRP3 signaling pathway, while other signaling pathways that can be potentially modulated by lycopene should be examined in future studies. Third, the effects of lycopene on EPC pyroptosis were examined by Western blot analysis, and future studies should consider the cellular immunofluorescence to consolidate our findings. Fourth, although our results have demonstrated the anti-pyroptosis effect of lycopene at the cellular level, further studies are necessary to examine its *in vivo* protective effects in the animal model of atherosclerotic cardiovascular disease.

## 5 Conclusions

Collectively, our study illustrates that lycopene may ameliorate EPC dysfunction and suppress pyroptosis induced by ox-LDL via the AMPK/mTOR/NLRP3 signaling pathway. This implies that lycopene could be a promising therapeutic approach for preventing atherosclerosis.
